# Evaluation of Influencing Factors on Tubal Sterilization Regret: A Cross-Sectional Study 

**DOI:** 10.22074/ijfs.2018.5272

**Published:** 2018-06-20

**Authors:** Shahideh Jahanian Sadatmahalleh, Saeideh Ziaei, Anoshirvan Kazemnejad, Eesa Mohamadi

**Affiliations:** 1 Department of Midwifery and Reproductive Health, Faculty of Medical Sciences, Tarbiat Modares University, Tehran, Iran; 2 Department of Biostatistics, Faculty of Medical Sciences, Tarbiat Modares University, Tehran, Iran; 3 Department of Nursing, Faculty of Medical Sciences, Tarbiat Modares University, Tehran, Iran

**Keywords:** Anxiety, Menstrual Cycle, Regret, Sexual Dysfunction, Tubal Ligation

## Abstract

**Background:**

The aim of this study is to evaluate the menstrual pattern, sexual function, and anxiety, and depression in women with poststerilization regret, and potential influencing factors for regret following tubal ligation (TL) in Iranian women.

**Materials and Methods:**

In this cross-sectional study, 166 women with TL were subdivided into two groups including women with poststerilization regret (n=41) and women without poststerilization regret (n=125). They were selected from a health care center in Guilan province (Iran) during 2015-2016. Menstrual blood loss was measured using the Pictorial Blood Loss Assessment Chart (PBLAC) and through a self-administered questionnaire. In addition, sexual function was assessed by the Female Sexual Function Index (FSFI), and psychological distress was measured by employing the Hospital Anxiety and Depression Scale (HADS). Student’s t test and Chi-square test were used to reveal the statistical differences between the two groups. We used logistic regression to determine the influencing factors associated with regretting sterilization.

**Results:**

Women with poststerilization regret had more menorrhagia (78 vs. 57.6%, P=0.03) than those who did not regret sterilization. A significant difference was found in sexual dysfunction in orgasm (P=0.02), satisfaction (P=0.004), pain (P=0.02), and total FSFI scores (P=0.007) between the two groups. Also, there was a significant difference between the two groups in anxiety, depression and total scores HADS (P=0.01). In the logistic regression model, age of sterilization [odds ratio (OR=2.67), confidence interval (CI): 1.03-7.81, P=0.04)], pre-sterilization counseling (OR=19.92, CI: 6.61-59.99, P<0.001), score of PBLAC (OR=1.01, CI: 1.004-1.01, P=0.001), the number of days of bleeding (OR=1.37, CI: 1.01-1.99, P=0.04), and the length of menstrual cycles (OR=0.91, CI: 0.84-0.99, P=0.03) were significantly associated with regretting sterilization.

**Conclusion:**

Complications due to sterilization are the main causes of regret; therefore, it is necessary to pay due attention to mentioning the probable complications of the procedures such as menstruation disorders, sexual dysfunction, and anxiety and depression in women during pre-sterilization counseling.

## Introduction

Permanent contraception method is a greatly desired and frequently used contraceptive option for women around the world who desire never to become pregnant (1). Tubal ligation (TL) is the most prevalent contraceptive method practiced in many countries, chosen by those women who no longer want children or have decided to limit the size of family (2). Despite the wide popularity of this method, more recent evidence suggests that some TL women may regret their decision after sterilization during the ensuing years (3).

 An increasing number of women have shown post-TL regret for their decision to undergo TL. Some studies have revealed that the prevalence of post-sterilization regret ranges from 0.9% to 26% (4, 5). Regret rates among sterilized women in Iran vary from 6 to 12.5% of all sterilized women (6). Definition of regret after sterilization may be different in the various studies, and this may bias the rates reported (7). The term “regret” is commonly associated with the feeling of sadness, pain, hurt, affliction, anxiety and displeasure, some authors have considered “clearly regretful” only for the women who obviously show their desire and intention to undergo surgery for reversal of sterilization (ROS) (8). The interval from post-sterilization regret and eventual request for ROS varies in different studies (9, 10).

Some factors such as sterilization age, the death of children, the number of children at the time of TL, remarriage, changes in socioeconomic status, and lack of information about surgical sterilization contribute to post-sterilization regret (3). If the factors associated with post-sterilization regret could be identified prior to TL, the feeling of regret by TL women could be prevented (7).

Although TL is particularly common in the developing than in the developed countries, recently the majority of studies on post-sterilization regret have been carried out on women in the developed countries (7). Research on sterilization regret in Iran is limited; hence, the overall objective of this pioneering study is to identify the potential influencing factors on regret following TL in Iranian women, and also to evaluate the menstrual pattern, sexual function, and anxiety, and depression in women regretting sterilization.

## Materials and Methods

In this cross-sectional study, first, a pilot study was conducted on 20 women. Then, using the appropriate formula with α set at 0.05 and 1-β at 0.95, it was found that a sample size of 40 women was needed for each group. This cross-sectional study was conducted on women (aged 20-40 years) undergone TL. They were selected from a health care center in Guilan province (Iran) during 2015-2016.

Satisfaction with TL was evaluated in response to questions such as “Do you think TL as a permanent method of birth control was a good choice for you?” Those who answered 'no' were further asked: “Do you regret for deciding to undergo sterilization?” If the answer was a consistent yes, then this group was considered to have regretted the decision (women regretting sterilization group). Further questioning continued to seek reasons for regret. This included interrogation regarding menstrual irregularities, depression and anxiety, sexual dysfunction, and having desire to have more children. The last question was: “Have you ever requested that your sterilization is reversed?” Possible responses included “no” and “yes” (11).

A total of 238 women were enrolled in the study; 166 women were eligible for inclusion, and 72 women were excluded from the study. The final analysis was conducted on 41 women regretting the decision, and 125 women not regretting the decision.

The inclusion criteria were free of any gynecological diseases, free of chronic diseases, include diabetes, hypertension, thyroid and cardiovascular diseases, not being in the postmenopausal period, not using antidepressants, not having the history of sexual abuse, not having the history of menstrual disorders before TL, not being a cigarette smoker, not having the history of operative gynecology except caesarean section and TL, and not doing breastfeeding.

We compared the distribution of demographic and obstetrical characteristics, menstrual disorder, sexual function, and depression and anxiety between the two groups.

This study was performed after obtaining approval from our Institutional Review Board (IRB # 1056668). All women participated voluntarily and provided a signed informed consent.

### Measures

Menorrhagia is defined as a Pictorial Blood Loss Assessment Chart (PBLAC) score of ≥100 (12). A validated PBLAC was also used for evaluating the Menstrual Blood Loss (MBL) (13). Participants used this chart to keep diaries on the amount of daily menstrual bleeding by marking the number of clots, and the amount of staining on each pad or tampon. Everyone completed their charts for one menstrual cycle, and all patients used the same sanitary products.

The participants’ sexual function was evaluated and compared by using the Female Sexual Function Index (FSFI) questionnaire. This standardized questionnaire is a validated, 19 items, self-administered, and a screening tool that measures six aspects of sexual function (desire, arousal, lubrication, orgasm, satisfaction, and pain). Each question describes the status of sexual function during the last 4 weeks. The full-scale score range is from 2.0 to 36.0, with higher scores associated with a lesser degree of sexual dysfunction (14). In this study, we used the Persian version translated by Mohammadi et al. (15). A score <3.3 in the desire domain, score <3.4 in arousal and orgasm, score <3.8 in satisfaction and pain, score <3.7 in lubrication, and total score <28 were considered as sexual dysfunction.

The Hospital Anxiety and Depression Scale (HADS) was used to assess depression and anxiety. The instrument has two subscales including anxiety (HADS-A) and depression (HADS-D). The HADS is a self-administered instrument consisting of 14 questions. The instrument has two subscales including anxiety (seven items) and depression (seven items). All items rate from 0 to 3. Sum scores <8 indicate normal range; scores 8-10 reflect mild alterations and scores ≥11 indicate clinical relevance of symptoms (16). A study on Persian version of the HADS has shown that this scale has a satisfactory reliability and validity for measuring psychological symptoms in Iranian patients (17).

The study was approved by the Tarbiat Modares Ethical Committee and all subjects signed a written informed consent.

### Statistical analysis

All statistical analyses were performed by the SPSS software (version 20.0, SPSS Inc., Chicago, IL, USA). Student’s t test and Chi-square test were used to reveal the statistical differences between the two groups, after adjusting for women’s age (at the time of data collection), age at the time of sterilization, partner’s age, education levels, BMI. We used logistic regression to determine the influencing factors associated with regretting sterilization. Women’s age (at the time of data collection), age at the time of sterilization, pre-sterilization counseling, PBLAC score, the number of days of bleeding days, the length of menstrual cycles, total score of FSFI, and total score of HADS were included in the regression analysis as continuous variables. Odds ratio (OR) at 95% confidence interval (CI) were also calculated for each factor. P<0.05 were considered to be statistically significant.

## Results

The mean duration of TL was 4.6 ± 1.2 years. The demographic and reproductive of participants are shown in Table 1. Both groups were not significantly different in terms of age (at the time of data collection), partner’s age, menarche age, BMI, parity, educational level, previous contraceptive use, and the method of delivery. There were significant differences in pre-sterilization counseling between the two groups. Of 166 women who completed the questionnaires, 34.9% did not receive any pre-sterilization counseling from a physician or a healthcare worker (Table 1).

Regret declined as the age of the TL women increased. There was a significant difference between women with poststerilization regret and those who did not regret sterilization. Post TL regret was found in those aged less than 30 years (age at the time of sterilization) and those above the age of 30 (P=0.01, Table 1). Our participants did not have a history of remarriage or the death of children.

### Menstrual pattern status in the two groups of study after adjusting

Table 2 displays the findings regarding the participants' menstruation disorders. There was a significant difference between the two groups in PBLAC score for menstrual loss between the two groups. The mean score of PBLAC was significantly higher in the women with poststerilization regret compared to their counterparts who did not regret sterilization (214.21 ± 116.08 vs. 126.24 ± 72.46, P<0.001) (Table 2). The women regretting sterilization had more menorrhagia (78 vs. 57.6%, P=0.03) than those who did not regret sterilization. There is a significant difference between the two groups in the length of menstrual cycles (P=0.005), and also in the number of days of bleeding (P<0.001, Table 2).

**Table 1:** Comparison of demographic and personal characteristics between two groups


Parameter	Regret n=41		Non-regret n=125		P value
	Mean ± SD	n (%)	Mean ± SD	n (%)	

Women’s age (Y)	36.06 ± 3.20		35.95 ± 4.40		0.10^**^
Partner’s age (Y)	40.51 ± 4.84		41.21 ± 4.13		0.36^**^
Age of menarche (Y)	12.78 ± 1.72		12.64 ± 1.23		0.57^**^
Age of sterilization (Y)					
≤30		19 (46.3)		30 (24)	0.01^*^
>30		22 (53.7)		95 (76)	
Parity	2.36 ± 0.58		2.32 ± 0.56		0.65^**^
BMI (Kg/m^2^)	28.00 ± 5.92		27.96 ± 4.62		0.97^**^
Education level					
Primary school		13 (31.7)		24 (19.2)	
Completed high school		12 (29.3)		50 (40)	0.20^*^
University		16 (39)		51 (40.8)	
Method of delivery					
Normal vaginal delivery		10 (24.4)		31 (24.8)	0.12^*^
Caesarean section		31 (75.6)		94 (75.2)	
Previous contraceptive method used					
Pill		4 (9.8)		7 (5.6)	
Condom		31 (75.6)		104 (83.2)	0.51^*^
Other^***^		6 (14.6)		14 (11.2)	
Pre-sterilization counseling					
No		35 (85.4)		23 (18.4)	<0.001^*^
Yes		6 (14.6)		102 (81.6)	


BMI; Body mass index, *; Chi-square test, **; t test, and ***; This category included withdrawal and natural family planning or the rhythm method.

**Table 2:** Comparison of changes in menstrual function between two groups


Parameter	Regret n=41		Non-regret n=125		OR adjusted(95% CI)	P value^*^
	Mean ± SD	n (%)	Mean ± SD	n (%)		

Menstrual cycle length (day)	25.34 ± 6.81		29.01 ± 5.94		0.89 (0.83-0.96)	0.005
Duration of bleeding menstrual (day)	7.41 ± 1.91		6.30 ± 1.35		1.64 (1.24-2.16)	<0.001
Menstrual irregularities		13 (31.7)		20 (16)	2.14 (0.91-5.05)	0.07
Menorrhagia		32 (78)		72 (57.6)	2.44 (1.04-5.69)	0.03
PBLAC score	214.21 ± 116.08		126.24 ± 72.46		1.01 (1.006-1.01)	<0.001


BMI; Body mass index, OR; Odds ratio, CI; Confidence interval, and *; P values are adjusted for women’s age (at the time of data collection), age at the time of sterilization, education levels, BMI.

### Sexual function status in the two groups of study after adjusting

Evaluation of the two groups by FSFI showed that all mean values were lower in the women with poststerilization regret. The differences of scores in the two groups were statistically significant in the domains of orgasm (OR= 0.68, CI:0.49-0.94, P=0.02), satisfaction (OR=0.59, CI:0.41-0.84, P=0.004), pain (OR=0.72, CI:0.54-0.95, P=0.02), and total FSFI scores (OR=0.88, CI:0.88-0.96, P=0.007) (Table 3).

The women regretting with poststerilization regret had more sexual dysfunction in the domains of satisfaction (48.8 vs. 30.4%, P=0.03), pain (48.8 vs. 28%, P=0.01), and total FSFI scores (63.4 vs. 40.8%; P=0.01) than the other group (data not shown).

### Anxiety and depressive status in the two groups of study after adjusting

The mean scores of anxiety and depression were found to be higher in the women with poststerilization regret compared to their counterparts who did not regret sterilization, and the differences between the two groups were statistically significant on anxiety scale (OR=1.14, CI:1.03-1.27, P= 0.01), depression scale (OR=1.14, CI:1.02-1.27, P=0.01), and total HADS scores (OR=1.09, CI:1.02-1.16, P= 0.01) (Table 3).

Of the women regretting sterilization, 61% (n=25) demonstrated elevated HADS anxiety scores (i.e. HADS anxiety subscale ≥11), and 17.1% (n=7) showed higher HADS depression scores (i.e. HADS depression subscale ≥11). Finally, 35 women (85.4%) in the women regretting sterilization group scored above the cut-offs (≥11) for both anxiety and depression (data not shown).

### Reasons for sterilization, regret, and reversal

The reason for requesting sterilization in the majority of women was the higher effectiveness of sterilization (36.8%) as compared to other methods. Other reasons were having enough children or having no desire for more children (35.5%), and unsatisfied with other contraceptive methods for their many side effects (27.7%).

**Table 3:** Scores and total scores for the domain subgroups of sexual function and HADS between two groups


Parameter	Regret n=41 (Mean ± SD)	Non-regret n=125 (Mean ± SD)	OR adjusted (95% CI)	P value^*^

FSFI
	Desire	2.83 ± 0.78	3.11 ± 0.76	0.66 (0.39-1.10)	0.11
	Arousal	3.16 ± 1.04	3.49 ± 0.89	0.70 (0.45-1.07)	0.10
	Lubrication	3.68 ± 1.28	4.06 ± 1.08	0.80 (0.56-1.14)	0.22
	Orgasm	3.55 ± 1.42	4.17 ± 1.13	0.68 (0.49-0.94)	0.02
	Satisfaction	3.82 ± 1.14	4.49 ± 1.13	0.59 (0.41-0.84)	0.004
Pain	3.81 ± 1.65	4.41 ± 1.19	0.72 (0.54-0.95)	0.02
Total score	20.87 ± 5.91	23.75 ± 4.52	0.88 (0.88-0.96)	0.007
HADS
	Anxiety score	11.39 ± 4.06	9.48 ± 3.67	1.14 (1.03-1.27)	0.01
	Depression score	7.97± 3.68	6.12 ± 3.49	1.14 (1.02-1.27)	0.01
	Total score	18.97 ± 6.75	15.56 ± 6.07	1.09 (1.02-1.16)	0.01


FSFI; Female Sexual Function Index, HADS; Hospital Anxiety and Depression Scale, BMI; Body mass index, OR; Odds ratio, CI; Confidence interval, and *; P values are adjusted for women’s age (at the time of data collection), age at the time of sterilization, education levels, BMI.

No significant difference was found between the women regretting sterilization and the women not regretting sterilization in reasons for requesting sterilization (data not shown).

When the women with poststerilization regret were asked to state reason(s) for regret, 43.9% (n=18) had both menorrhagia, anxiety and depression, 19.5% (n=8) reported having sexual problems, menorrhagia, and anxiety and depression after the operation, 14.6% (n=6) had anxiety and depression, 12.2% (n=5) simply wanted to have another child, and about 9.8% (n=4) regretted the operation because it brought about only menorrhagia.

Requesting ROS after TL was 3% (5 women). The reasons for requesting ROS after TL involve both menorrhagia, and anxiety and depression (n=2), both sexual problems and menorrhagia (n=1), desire for having more children (n=1), and only menorrhagia (n=1). Age at the time of sterilization can affect desire for ROS. Women younger than 30 years at the time of sterilization were more likely to request reversal than those who were above 30 years old (80 vs. 20%, P=0.02) (data not shown).

There were significant differences in pre-sterilization counseling between the women requested reversal and those who did not. Of the 5 ROS women, nobody did receive any pre-sterilization counseling from a physician or a healthcare worker (P=0.005) (data not shown).

Finally, in order to build a prediction model and to find the most important factors predicting with poststerilization regret, we used a logistic regression model in a backward manner. The results of fitting the logistic regression model to the data (Table 4). Only significant results are presented in Table 4.

**Table 4:** Logistic regression analysis of 166 women for regretting sterilization


Parameter		OR (95% CI)	P value^*^

Age of sterilization (Y)			
	≤30	2.67 (1.91-7.81)	0.04
	>30	1^†^	
Pre-sterilization counseling			
	No	19.92 (6.62-59.90)	<0.001
	Yes	1^†^	
PBLAC score		1.01 (1.004-1.07)	0.001
Number of days of bleeding		1.37 (1.01-1.99)	0.04
Length of menstrual cycles		0.91 (0.83-0.99)	0.03
Constant		0.012	0. 01


OR; Odds Ratio, CI; Confidence interval, †; Reference category, *; P value logistic regression, and women’s age (at the time of data collection), age at the time of sterilization, parity, pre-sterilization counseling, PBLAC score, number of days of bleeding, length of menstrual cycles, total score of FSFI, and total score of HADS were included in the regression analysis as continuous variables. Only significant results are presented.

In the logistic regression model, age of sterilization (OR=2.67, CI: 1.91-7.81, P=0.04), pre-sterilization counseling (OR=19.91, CI: 6.62-59.90, P<0.001), score of PBLAC (OR=1.01, CI: 1.004-1.07, P=0.001), and the number of bleeding days (OR=1.37, CI: 1.01-1.99, P=0.04) were significantly associated with poststerilization regret. However, the length of menstrual cycles (OR=0.91, CI: 0.83-0.99, P=0.03) was negatively related to regretting sterilization regret (Table 4).

## Discussion

Tubal ligation is chosen by the women who have decided to limit the size of their families or those who are sure that no longer want to have children (18). The goal of this study was to evaluate the menstrual pattern, sexual function, and anxiety and depression, in women regretting sterilization, and also identify the potential influencing factors for regret following TL. To our knowledge, none of the several recent studies on Iranian population have investigated the influencing factors for regret after TL.

The present results indicated that women with poststerilization regret were more likely to experience an increase in menorrhagia when compared with the other group. We found a significant increase in PBLAC score for menstrual blood loss in the women regretting sterilization when compared with the other group. Also the women regretting sterilization were more likely to experience a shortening of the duration of menses and an increase in the number of days with bleeding. The term “Post-TL Syndrome” (PTLS) has been used variously to include menstrual disorders, dysmenorrhea, premenstrual distress, and miscellaneous other conditions like menopausal syndrome, feeling of regret, and need for recanalization (19). Wilcox et al. (20) reported that menstrual irregularities were not more prevalent among the women regretting their sterilization than those not regretting their sterilization. Malhotra et al. (21) mentioned that menstrual irregularities and dysmenorrhea did not influence regret.

Our findings suggest that menorrhagia were more common in the women regretting sterilization in comparison to the women not regretting sterilization. The relationship between regretting sterilization and menstrual disorders is a complex process influenced by multiple factors including psychological and cultural conditions, as well as behavior, ethnicity, climate, and religion.

It was revealed that regret following TL may be an influencing factor of women’s sexual dysfunction. In the present study, the prevalence of FSD in the women regretting TL was 63.4% in comparison with 40.8% in the other group. Warehime et al. (22) suggested a relationship between sexual dysfunction and post-sterilization regret in women with TL. Shah and Hoffstetter (23) reported that TL had a positive impact on sexual function unless women regretting sterilization.

“Making a decision about sterilization is difficult for both women and men, as it means ending fertility. As negative biological and psychological issues may occur after vaginal surgeries including loss of sexual function, the same negative effects after TL could be expected”. The negative effects of PTLS on general health status and the sexual function have not been described yet (24). “In societies such as Iran, the picture a woman holds of herself is to a high degree dependent on her fertility and motherhood ability. It conveys to women feelings of satisfaction, perfection, and value. Despite the women’s volunteer participation in the sterilization programs, the cultural factors, which are rooted in their deep unconscious layers, seem to produce in some of them an unattractive and imperfect picture after a while; this can be reflected in the appearance of sexual dysfunction and regret from this operation” (25).

The results of the present study showed that anxiety and depression were more common in the women regretting sterilization in comparison to the other group. Kelekçi et al. (26) reported that anxiety and depression scores in the women regretting sterilization were significantly higher than in the control group. Regret following TL may contribute to higher anxiety and depression scores after the procedure, and may pointedly affect the psychological health of women. In addition, they suggested that women with high anxiety and depression scores must be given time to consider their decision on fertility state, and should be provided psychological support during this time. A high preoperative anxiety and depression score may be an essential factor in future post-TL regret and high anxiety and depression scores (27). Counseling before sterilization, especially in younger women, needs to cover alternative birth control methods, irreversibility, and failures of the procedure. It should future elicit information about the couples’ psychosocial and marital dynamics, the somatic or psychological symptoms, as well as women’s sexual history and menstrual history (28).

The psychological status of women prior to the sterilization technique may be important in predicting future satisfaction and success of an operation. Depression is one of the most prevalent illnesses in women, which significantly affects their quality of life. It has been thought that anxiety and depression are related to cyclical hormonal changes, which may be affected by TL (26).

In the present study, the prevalence of requesting ROS after TL was 3%. Age at the time of sterilization can affect desire for ROS. Women younger than 30 years old at the time of sterilization were more likely to request reversal than women above the age of 30 (80 vs. 20%). There were significant differences in pre-sterilization counseling between the women requested reversal versus those not requested reversal. From 5 ROS women, nobody did receive any pre-sterilization counseling from a physician or healthcare workers.

Previous studies have indicated several factors predicting desire for ROS (3). Age at the time of sterilization can affect desire for ROS. Women with TL at younger ages were significantly more likely to experience regret than older TL women (7). Rogayeh et al. (6) reported that 2.7% of the sterilized women requested a reversal surgery. Overall, recent evidence suggests that the younger the women are when they undergo TL, the more likely they are to regret their decision, to request for information regarding ROS, or to obtain a ROS (7).

Young age at sterilization, pre-existing emotional disorders, new marriage, and a history of unreliable contraceptive method increased the likelihood of a request for ROS. Women requesting TL should be advised that TL is permanent if no further tubal surgery is performed and that ROS is feasible but involves expensive operation (2).

In this study, we aimed to examine various parameters that might predict the incidence of regretting sterilization in women with TL. Age at the time of sterilization, pre-sterilization counseling, score of PBLAC, and the number of days of bleeding was evaluated as predictors for regretting sterilization. In addition, the length of menstrual cycles was negatively associated with regretting their decision. We found a significant difference in the age at the time of sterilization under 30 years between the women regretting versus those not regretting their sterilization. Young age at sterilization was the strongest predictor for post-sterilization regret (3, 7). Rogayeh et al. (6) reported that post-sterilization regret rate did not increase for the women under 30 or younger when sterilized.

Additionally, the other factor significantly associated with regret is counseling before sterilization (29). This result supports the recommendation that women should be fully informed about the TL procedure and its complications, and have access to other contraceptive options before being sterilized. If factors that lead to regret could be identified prior to sterilization, some of this regret may be prevented (7). It was revealed that there were significant differences in pre-sterilization counseling between the two groups. The women regretting sterilization group had lower pre-sterilization counseling than in the other group. In our study, pre-sterilization counseling was only reported by 14.6% of the women regretting sterilization. With respect to personality and adaptability differences in facing the changes, pre-sterilization counseling has an important role to play in psychological and psychosexual aspects of women health promotion (6). This finding is consistent with the results of many similar studies conducted in other countries (6, 21). Allyn et al. (28) reported a positive correlation between the degree of contentment with pre-sterilization counseling and the level of satisfaction with being sterilized. It was also found that only 11.3% of under 25 women at the time of surgery completely regretted their sterilization. The ethnic backgrounds of the participants and the number of living children at the time of TL had no significant effect on their satisfaction with sterilization.

More knowledge about the potential influencing factors, which have a strong association with regretting the sterilization decision and are easily identifiable before sterilization, is definitely valuable in counseling to avoid regrets. Motivators who may not be doctors in our society need to be informed of the influencing factors prior to counseling women for TL (21).

Although post-sterilization regret is hardly avoidable, certain groups such as women under 30 years need specific counseling before taking such a major decision like TL (21).

There are potential limitations to consider in interpreting the results of this study. First, expressing post-sterilization regret is generally an attitudinal measure for which there is no standardized definition. For this reason, these rates may overestimate true sterilization regret. Second, we could not assess the method of TL because women did not have information about the type of sterilization.

## Conclusion

The results showed that complications due to sterilization are the main causes of post-sterilization regret. Therefore, it is necessary to pay due attention to mentioning the probable complications of the procedure such as menstruation disorders, sexual dysfunction, and anxiety and depression in women during the pre-sterilization counseling; post-sterilization counseling is also encouraged for increasing satisfaction rate in these volunteers.
